# Effects of music therapy in the treatment of children with delayed speech development - results of a pilot study

**DOI:** 10.1186/1472-6882-10-39

**Published:** 2010-07-21

**Authors:** Wibke Groß, Ulrike Linden, Thomas Ostermann

**Affiliations:** 1 Nordoff Robbins Centre of Music Therapy, Ruhrstrasse 70, 58452 Witten, Germany; 2 Department of Music Therapy, Community Hospital Herdecke, Gerhard-Kienle-Weg 4, 58313 Herdecke, Germany; 3 Center of Integrative Medicine, University of Witten/Herdecke, Gerhard-Kienle-Weg 4, 58313 Herdecke, Germany

## Abstract

**Background:**

Language development is one of the most significant processes of early childhood development. Children with delayed speech development are more at risk of acquiring other cognitive, social-emotional, and school-related problems. Music therapy appears to facilitate speech development in children, even within a short period of time. The aim of this pilot study is to explore the effects of music therapy in children with delayed speech development.

**Methods:**

A total of 18 children aged 3.5 to 6 years with delayed speech development took part in this observational study in which music therapy and no treatment were compared to demonstrate effectiveness. Individual music therapy was provided on an outpatient basis. An ABAB reversal design with alternations between music therapy and no treatment with an interval of approximately eight weeks between the blocks was chosen. Before and after each study period, a speech development test, a non-verbal intelligence test for children, and music therapy assessment scales were used to evaluate the speech development of the children.

**Results:**

Compared to the baseline, we found a positive development in the study group after receiving music therapy. Both phonological capacity and the children's understanding of speech increased under treatment, as well as their cognitive structures, action patterns, and level of intelligence. Throughout the study period, developmental age converged with their biological age. Ratings according to the Nordoff-Robbins scales showed clinically significant changes in the children, namely in the areas of client-therapist relationship and communication.

**Conclusions:**

This study suggests that music therapy may have a measurable effect on the speech development of children through the treatment's interactions with fundamental aspects of speech development, including the ability to form and maintain relationships and prosodic abilities. Thus, music therapy may provide a basic and supportive therapy for children with delayed speech development. Further studies should be conducted to investigate the mechanisms of these interactions in greater depth.

**Trial registration:**

The trial is registered in the German clinical trials register; Trial-No.: DRKS00000343

## Background

Music therapy is an established form of creative art therapy. By using music as a specific medium of communication and expression and adapting it to the individual resources and abilities of the patient, music therapy can be beneficial in activating and supporting mental and psycho-physical recovery. Several systematic reviews have shown the effects of music therapy in different clinical and therapeutic settings, such as for the treatment of psychiatric diseases like schizophrenia or schizophrenia-like illnesses [1], psychosis [2], neurological diseases like multiple sclerosis [3], dementia [4], or for the treatment of anxiety and pain [5].

In addition to these therapeutic fields, music therapy can also be applied in the treatment of developmentally delayed children. Already in 1995, Aldridge et al. illustrated the use of music therapy in children with developmental delay [6]. In another study, Duffy and Fuller (2001) found that an 8-week music therapy intervention in social skills development in moderately disabled children resulted in an increment in terms of turn-taking, imitation, and vocalization [7]. Perry (2003) found direct relationships between the level of communication skills and elements of musical interaction in children with severe and multiple disabilities [8]. Finally, Kim et al. (2008) demonstrated the effects of music therapy on joint attention behaviours in preschool children with autism in a randomized controlled study [9].

Developmental delay often accompanies delayed speech development. Speech development is an important predictor for later problems, such issues with reading and spelling, among other learning difficulties. Gallagher [10] found that "studies of children with language impairment have reported emotional and behavioural problems in 50-75% of that population". According to Sallat [11], different authors describe children with delayed speech development as being highly at risk of other cognitive, social-emotional, and school-related problems.

However, definitions of speech development disorders still differ greatly and data on the prevalence of delayed speech development actually range from 4 to 40% [12]. Grimm et al. [13] detected substantial speech development disorders in 10% of children between the ages of 4 and 5 in Bielefeld, Germany, and suspected speech development disorders in 20%. General textbooks quote the epidemiology of specific speech development disorders as being between 3 and 5%; however, this is without a traceable background [14]. According to a study in Bavaria, 22.5% of tested children showed at least one problem in various tested areas that required speech therapy [15].

Although there are considerable epidemiological variations due to the definition of developmental speech delay, its prevention is a challenging social issue. In this respect, it is of vital importance to apply therapies that are able to support the salutogenetic capacities of the child with the aim of enhancing his or her speech development as early as possible.

According to findings of Aldridge [6] and Schumacher [16], music therapy is an approach that may facilitate significant advances in speech development and communication skills, particularly in children with autism. Additionally, Lathan-Radocy [17] described different ways and methods of working with speech and language impaired children by engaging them in music therapy. Finally, several case studies found positive effects of music therapy on speech development in children [18-20].

Based on these findings, this study aims at examining the effectiveness of music therapy on a child's verbal reasoning abilities. Furthermore, we wanted to investigate whether experiencing musical structures, such as rhythm or strophic forms, and improvising with a music therapist could stimulate a child's ability to understand sentences, as well as encourage his or her interest in communicating with others.

## Methods

### Study design

This observational pilot study was conducted in the Department of Music Therapy at Herdecke Community Hospital between 2006 and 2008. We chose an ABAB reversal design with alternations between music therapy and no treatment with an interval of approximately eight weeks between the blocks. Before and after the music therapy blocks, a speech therapist and a psychologist, who were both blinded to the conditions and timing, tested the children using validated speech and nonverbal developmental tests. The study was approved and accepted by the ethics commission at the University Witten/Herdecke (application number: 115/2006) and is registered in the German clinical trials register (Trial-No.: DRKS00000343; http://www.drks.de).

### Participants and inclusion criteria

All children were recruited via announcements in integrative and regular nursery schools. Parents contacted the music therapy department at the hospital by phone and after the first selection, 39 children aged 3.5 to 6 years, all with German as their native language, were eligible to participate in the study. Participants had to pass a medical examination and take a speech test to check whether they had a specific developmental speech disorder (ICD-10-Codes: F80.1, F80.2, F83). They also had to score below 50 out of 100 points in the phonological short-term memory test for non-words (German: PGN) and in one other subscale of the applied speech test SETK 3-5 (see "Test instruments" section for a detailed description). Children diagnosed with autism or muteness and/or a speech development disorder due to any organic causes (e.g. deafness) were excluded, as well as those children who had previous experience with music therapy.

**Figure 1:**
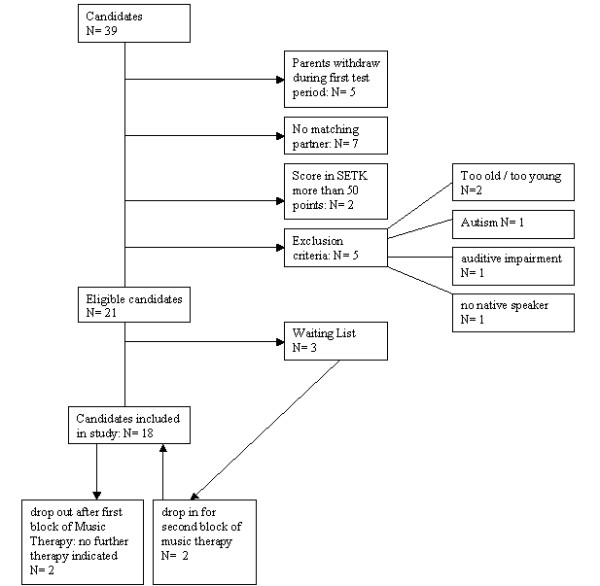
Consort diagram of the selection process

As a result, a total of 18 children (6 girls; mean age: 4.3 ± 0.5 years) were selected to participate in the study (see Figure 1 for the consort diagram of the study). None of the participants were physically disabled and all were able to move and act independently.

Parents or guardians provided written informed consent for their child's participation in the study before enrolment of the study. This included a discussion of the music therapist on the nature and purpose of the proposed music therapy treatment and its potential risks and benefits. Parents were also given the opportunity to ask questions to elicit a better understanding of the music therapy treatment. Accompanying therapies, like speech therapy and early intervention programs, continued uninterrupted due to ethical considerations.

### Setting

Children received music therapy on an out-patient basis at the Department of Music Therapy at Herdecke Community Hospital. The participants were brought to music therapy sessions by their parents, but entered the music therapy room alone when possible. Single therapy sessions had a mean duration of 25 minutes and were provided by a therapist and co-therapist together. To meet the quality criteria of the hospital, the two music therapists had to have a master's degree in music therapy and sufficient clinical experience (a minimum of two years) in their field.

### Music therapy

This study applied the concept of creative music therapy based on the Nordoff-Robbins approach [18]. Both patient and therapist were active in singing and making music with percussion instruments (i.e. bells, drums, pentatonic tone bars, shakers, reed horns, and lyres) and a piano. Songs specifically composed for playtime and that dealt with the child's interests, such as hide-and-seek songs or songs about animals, completed the therapeutic spectrum. Individual themes and musical developments thus emerged for each individual child; some wanted to sing and dance, others wanted to be sung to, and some wanted to play an instrument on their own. According to this individualized approach, the improvised music was oriented at the musical and vocal expressions of the child and therefore played the central role of the therapy.

### Test instruments

#### SETK 3-5

The speech development test for children aged three to five years (SETK 3-5) is the first standardized German language test to examine speech abilities in children of this age group with an immediate correlation between linguistic and auditory memory performance. With Cronbach's alpha values between 0.62 and 0.89 and an inter-rater reliability of 90.1%, the test yields a "reliable and valid description of receptive and productive speech abilities in children" [21] and covers three areas of speech development, divided into three categories with five subtests:

1. Children's understanding of speech (subtest "understanding sentences"; abbrev.: *VS)*

2. Speech production (subtest "generation of morphological rules"; abbrev.: *MR*)

3. Memory of speech, with a focus on abilities of speech processing, not on qualities of pronunciation (subtest "phonological memory for non-words"; abbrev.: *PGN*; subtest "memory for sentences"; abbrev.: *SG*, and subtest "memory for word sequences"; abbrev.: *GW*)

#### SON-R

The SON-R 2 1/2 - 7 is an individual intelligence test which does not require the use of spoken or written language [22]. It is especially suited for children with problems in the area of language and verbal communication and is comprised of six subtests of about 15 items that address the dimensions activity patterns and cognitive structures.

Activity patterns include the scales "categories (abstract thinking, organizing principles)", "analogies (abstract thinking)" and "situations (concrete thinking)". The scales "mosaics (spatial comprehension)", "puzzles (concrete thinking)", and "drawing structures (spatial comprehension)" represent the dimensions of the child's mental structure. Norm tables for monthly age groups enable the transformation of raw subtest scores into normalized standard scores. The total test results are represented as IQ scores and reference ages. IQ scores between 89 and 110 are average, 79 to 88 are below average, 68 to 78 are borderline, and IQ values below 67 indicate an intellectual deficit.

With a Cronbach's alpha value of 0.90 and a test-retest reliability of r = 0.79 for the overall IQ score, the reliability of the test is sufficient.

All psychometric tests were conducted by speech therapists and a psychologist blinded to the conditions and timing.

#### Music therapy assessment scales

All sessions were videotaped for analysis and consecutively assessed in detail after each session independently by the therapist and co-therapist. Nordoff-Robbins assessment scales were used to evaluate developments in music therapy for the first and last session of each music therapy block respectively. Scale I assessed the "child-therapist relationship in musical activity" (CTR) and Scale II assessed "musical communicative ability" (MCA); both were measured on a 10-point scale where 0 denotes lowest values and 10 denotes highest values in the respective categories. Inter-rater reliability values showed high agreement rates within the range of one point of 82% in Nordoff-Robbins therapists ratings [18].

### Statistical analysis

Data were analyzed using intention-to-treat analysis (ITT). Missing values were imputed using the method of last observation carried forward with the following rules: a missing value before a block of music therapy or no treatment was replaced with the corresponding pre-session value. A missing value after a session was replaced with the according pre-session value.

The Friedman test was used to analyze the effect of music therapy over the course of time and the Wilcoxon rank-sum test was used for baseline comparisons (T_0_) and final measurements after the last therapeutic session (T_4_). We judged p £ 0.05 as significant and p between 0.05 and 0.1 as a trend. To quantify the outcome we also calculated effect sizes for all scales between T_0 _and T_4_. According to Cohen [23], effect size values between 0.2 and 0.5 are indicative of a small effect; values between 0.5 and 0.8 denote a medium effect and values greater than 0.8 indicate a large effect size.

## Results

### SETK

Mean SETK scores yielded the following results: "phonological memory for non-words" (PGN) and "understanding sentences" (VS) revealed distinct upward trends, whereby increases were more pronounced during periods with music therapy. These two parameters showed mostly parallel development. These two subtests PGN and VS showed a particularly steeper increase after music therapy blocks (T0-T1/T2-T3) compared to waiting periods (T1-T2/T3-T4). "Memory for sentences" (SG) also improved distinctively, starting from a very low level. A second boost was registered after the second waiting period (see Table 1).

**Table 1:** Changes in the course of time and overall effect sizes in SETK subscales and SON-R outcome measures

	**T**_**0 **_**Mean ± Sd**	**T**_**1 **_**Mean ± Sd**	**T**_**2 **_**Mean ± Sd**	**T**_**3 **_**Mean ± Sd**	**T**_**4 **_**Mean ± Sd**	Fried-man-Test	**Wilcoxon-Test (T**_**1**_**-T**_**4**_**)**	**Effectsize(T**_**1**_**-T**_**4**_**)**
**SETK**								
PGN	37.4 ± 10.2	40.0 ± 11.0	41.5 ± 14.4	44.7 ± 16.3	47.0 ± 14.5	<0.0005	0.002	0.45
VS	37.4 ± 9.37	41.9 ± 12.7	43.7 ± 15.4	47.0 ± 16.3	48.2 ± 16.1	0.001	0.001	0.39
SG	30.4 ± 11.4	36.9 ± 12.4	35.2 ± 14.3	36.9 ± 13.5	39.9 ± 22.1	0.076	0.028	0.61
MR	40.8 ± 12.6	42.6 ± 10.7	41.6 ± 15.2	47.9 ± 17.6	46.9 ± 16.5	0.116	0.054	0.19
GW	3.0 ± 0.7	2.9 ± 1.3	3.1 ± 1.2	3.1 ± 1.1	3.2 ± 1.1	0.077	0.480	0.03
**SON-R (ref-age)**								
HS	3.2 ± 1.0	3.6 ± 1.0	4.0 ± 1.0	4.0 ± 1.2	4.2 ± 1.2	<0.0005	<0.0005	0.61
DS	3.5 ± 1.2	3.8 ± 1.3	4.0 ± 1.0	4.3 ± 1.2	4.3 ± 1.3	<0.0005	0.002	0.31
IQ	3.3 ± 1.1	3.7 ± 1.1	3.9 ± 1.0	4.1 ± 1.2	4.3 ± 1.3	<0.0005	<0.0005	0.57

"Generation of morphological rules" (MR) also increased after music therapy blocks while decreasing during waiting periods. "Memory for word sequences" (GW) was the hardest factor to assess. It was measured in different units compared to the rest of the parameters and in addition it is only measured in children aged four and older, so that the number of test results gained in this instance is far smaller. All general developments (T0-T4) for the five subtests of the speech test SETK revealed a definite increase. Over the entire study period (T0-T4), parameters PGN and VS showed statistically significant results (p < 0.001), which suggest that music therapy may have an effect on the development of phonological memory and understanding sentences. Phonological memory (PGN) indicated statistically significant results during the first waiting period (T1-T2 p = 0,008) and also after the second block of music therapy (T2-T3 p = 0,001; see Table 1).

### SON-R

The three parameters of SON-R (cognitive structures (DS), action patterns (HS), and IQ increased significantly in the study course (DS p = 0,001; HS p < 0,001; IQ p < 0,001). The parameter of cognitive structures (DS) showed higher mean scores compared to activity patterns (HS) at time T0. It is interesting to note that DS and HS differed by several points at first (T0, T1) but later converged after the first interval (T2) to almost identical levels. The scores diverged again after the second music therapy intervention (T3), although this time on a higher level. The scores finally converged after the second interval (T4) (see Table 1).

### Difference between age and biological age

**Figure 2:**
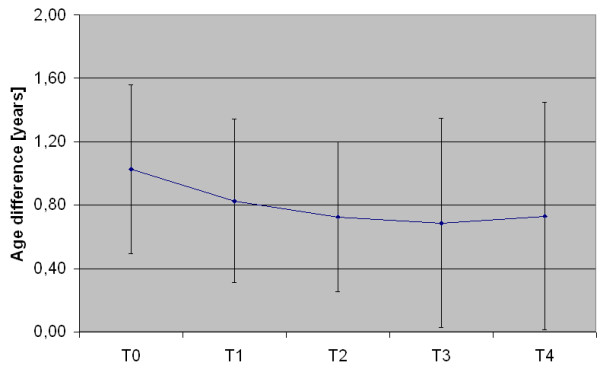
**Difference of developmental age (age IQ) to biological age over the study period**. Error bars denote the 95% confidence interval

Analysis of reference age revealed that the developmental age of the children in the course of music therapy interventions converged more and more towards their biological age. The difference was reduced from approximately one year at baseline to seven months at the end of therapy. Moreover, the variance in their developmental age increased meaning that some of the children corresponded with their biological age at the end of the study, while other children demonstrated a development approaching their biological age. The complete development over the course of time is shown in Figure 2.

### Nordoff-Robbins Scales

**Figure 3:**
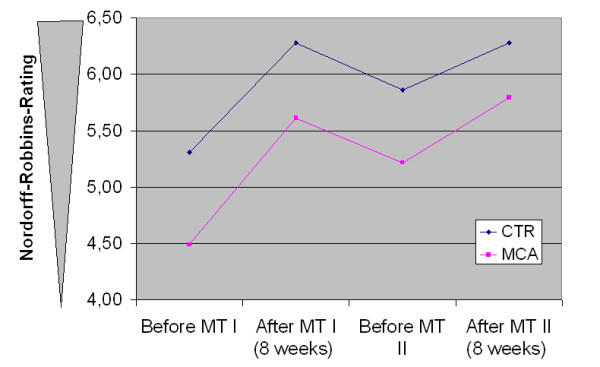
Development of Nordoff-Robbins rating over the study period

Ratings according to the Nordoff-Robbins scales showed distinctly significant changes. Both parameters CTR for child-therapist relation and MCA for musical communicative ability increased after the first music therapy block (T2), then subsequently decreased (T3), and then finally reached the previously achieved higher level after the second music therapy block (T4). MCA showed a slightly higher increase (Figure 3).

### Compliance

The compliance of the children was a major problem in the developmental tests, particularly in completing the SETK-questionnaire. While the SON-R was well accepted by the children and Nordoff-Robbins items were scored by the therapist, completing the SETK with the children was difficult, yielding to incomplete items in the SETK subscales (between 14% (VS-Scale) and 36% (GW-Scale) of the items). However, the imputation of missing values reduced these rates to a mean level of 10% remaining incomplete items.

## Discussion

This study is the first to provide valid information about the effects of music therapy in children with developmental speech delay. It was found that music therapy had an effect on fundamental qualities of speech development and resulted in significant improvements in phonological memory and the children's understanding of sentences. Furthermore, a positive shift in the memory of sentences and generation of morphological rules was observed. In particular, the difference between the developmental age and biological age of the children decreased significantly. These results were accompanied by a clinically significant effect of music therapy on the child-therapist relationship and musical communicative ability, as measured by the Nordoff-Robbins scales.

### Initial effect

Empirical observations frequently described the phenomena of an "initial effect" of music therapy on speech development (i.e. that music therapy seemed to stimulate the speech development of developmentally delayed children even after a few music therapy sessions). In accordance with these findings, results from this pilot study illustrate which aspects of speech development are influenced specifically by music therapy and show to what extent an initial effect may be detected. Results show improvements in the development of speech and cognitive abilities even after the first block of music therapy treatment. Children may benefit specifically in the areas of relationship and communication as shown by the rating according to the Nordoff-Robbins scales.

It is assumed that a newly acquired ability, such as a basal social experience, has an initial effect on the further development of the sense of self-perception and the perception of others. Furthermore, it influences self-awareness and emotional mood. This 'snow ball effect' could possibly explain the relatively fast positive development of the participants. Since communication is a basic human need, it can be further assumed that the provided individual communication model 'music therapy' was simply claimed by the participants after a successful introduction and was also maintained.

### Speech Development

The changes in phonological memory (PGN) and the understanding of sentences (VS) increased significantly with a parallel slope. Test results suggest that music therapy interventions may initially provide a boost in the development of these skills. Abilities covered by the subscale PGN are obviously related to prosodic abilities [24]. We believe that the improvements occur because music therapy addresses listening, perception, processing, and the memorizing of sounds and musical structures. This corresponds to a study by Jungblut et al. [25], who reported positive improvements in speech development in patients with aphasia due to music therapy. Here, prosody was one of the parameters showing substantial progress. This corresponds with the theoretical considerations of Grimm [24], who regards prosody as a defining aspect of speech processing and language acquisition.

The improvement in the VS subscale is underlined by the empirical observations we made in the study. In the beginning, most of the children we worked with had difficulty focusing their attention with hand-eye-coordination and the concentration on a joint activity with the therapist. During music therapy sessions, most of the children enhanced their concentration and were increasingly able to direct their eyes and concentration to a joint activity with the therapist and to playing an instrument. The improvement in the Nordoff-Robbins scales especially underlines these observations. Children benefit specifically in the areas of forming relationships and enhancing their communication skills. Parents, speech therapists, and teachers reported that the children started to communicate more frequently and started to have more social contacts. According to a deeper single case analysis of two children in our study, we found that one important element in achieving linguistic understanding is the ability to relate to another person [26]. Moreover, Grimm [24] describes three areas of so-called anticipatory abilities that are essential even in infants for acquiring speech: social cognition, perception, and cognition. These include the abilities to direct attention to objects and events, to differentiate between them, and to remember the differences. In addition, he determined that constructing a common point of focus has proved to be especially important in acquiring language skills.

The scores measured for SG also show a distinct increase over the study period. This may be explained through the inherent experience of structure and perspective in the process of active music making with the therapist. Studies of infants' abilities to perceive speech found that infants prefer well-structured speech patterns to less well-structured ones [24]. Perception and grasp of structures seem to be important skills for acquiring speech. Form and general structure of a sentence must be understood in order to grasp the entire meaning. These qualities are exercised and targeted all the time when making music. They may be shortened or expanded step by step and thus be adapted to the individual making the music and his or her abilities.

It is interesting to note that the parameter "generation of morphological rules" shows improvements after music therapy blocks. This parameter, quite unrelated to music at first sight, seems to address recognition and understanding of structures, which is continuously practised in active music-making. Deficits in generation of morphological rules are considered as particularly distinct and obstinate. Music therapy may give support to the development of this ability.

Rhythmic-prosodic abilities seem to be central for acquiring language. Again, Grimm describes that children with developmental speech delay frequently display considerable difficulties in the rhythmic-prosodic area. An impaired ability to grasp the totality of prosodic structures means that larger parts of the working memory must be relied upon, thus limiting the amount of working memory available for the understanding and processing of language. Training a child's reproduction capabilities of phonologic (and therefore also of prosodic) structures could provide significant support in the child's development of language abilities [24].

Aldridge [27] emphasized the importance of rhythmic structures and abilities for infantile development: "rhythm plays a central co-ordinating role in the organization of human perception and action, and for the developmentally delayed child, a controlled - yet flexible - rhythmic structure found in musical playing seems to be an island of stability from which new initiatives can take place." According to Trevarthen & Aiken [28], music therapy from a neuropsychological point of view may support human communication skills that are organized rhythmically in accordance with neurological processes. Thus, active, creative music therapy works immediately with the contact and communication between the improvising participants. In such a setting, the integration of several senses, like hearing or seeing, motor abilities, and emotion, is of vital importance [29] because both verbal communication and joint musical improvisation require a meaningful integration of these senses. Thus, music therapy may offer a specific space to test and develop various senses on a level appropriate to the child's individual abilities and speed.

From a music therapy perspective, Neugebauer [20] relates the steps of language development to musical qualities and concludes that music therapy works on those musical qualities and speech development can thus be enhanced. These findings are underlined by Papousek's research on infants [30]. She analyzed mother-infant interaction and its relation to musical parameters, even if the use of the musical metaphors has to be taken into account critically [29]. This might be an explanation of why music therapy can be effective in children with communication disorders. The therapeutic processes that took place in our study have been described comprehensively in the case report of two patients [26].

### Cognitive Development

The non-verbal development test SON-R produced encouraging results. The SON-IQ of the entire group rose significantly and the difference between developmental age and biological age of the children decreased significantly. Even here our study showed an initial effect. For a majority of children it may be assumed that their intelligence potential had not been fully exploited prior to the study. In music therapy we see many children who seem to have no experience whatsoever with symbolism, imagining fantasy stories, or playing with sounds. At first these children seem to soak up our imaginative-musical proposals before they start to develop and share their own creative potential. Music therapy thus seems to evoke and reveal unused potential. During music therapy, children seemed to access their potential and were even able to adapt it to another setting such as in the test situation. This corresponded to a detailed analysis by Rittelmeyer [31], who emphasized the impact of creative abilities in the neurological, cognitive, and emotional development of children.

An analysis of the two subtests for cognitive structures and action patterns is also of interest. At first, the two scales diverge, converge closely after the first waiting period in order to diverge again, and approach each other on a significantly higher level at the end of the study (see Figure 2). Cognitive structures are the first subtests to advance. Action patterns keep up with the development during the waiting period. Again an initial effect of music therapy can be assumed in this case. It is remarkable that action patterns converge to the similar level with cognitive structures. Cognitive structures and action patterns are nearly integrated at that point. A child cannot do much with certain cognitive abilities without knowing how to use them actively. An integration of thinking and doing appears to be indispensable for the meaningful use of cognitive abilities. Music therapy may therefore provide an important contribution to the promotion of integrated thinking and doing and may reveal a child's hidden potentials.

### Limitations

Although the large effect sizes in the present study point to a potential impact of music therapy, the small number of participants in the study should be mentioned as an important limiting factor. The discussion of whether observational studies tend to overestimate the effects of a therapy compared to the results of controlled clinical trials is still vital [32] and the call for randomized controlled trials (RCTs) has already reached the borders of music therapy [33]. Due to organizational and structural aspects we abstained from carrying out a RCT in this case. However, other researchers recommend more naturalistic, observational studies of patients in psychotherapy [34] and, with regards to external evidence, our findings give an impression of the real world effectiveness of music therapy. In order to verify the test results, further studies with a higher number of participants should be conducted to underline and specify the effects found in this setting [35,36].

As there is no control group or a specific control condition we are of course aware that the changes observed here might not be attributed to the music therapy applied. It could, for instance, simply be the intervention itself associated with a high amount of care giving or the attention given to the kids. A further point is that the children might have improved their behaviour simply in response to the fact that they were being studied. This is also known as the Hawthorne effect [37]. In his study on the effects of background music on quality of sleep in elementary school children, Tan also suggested, that children might be responding to music therapy treatment due to their awareness of participation [38].

Also, unblinding might be a potential source of bias. According to a study of Noseworthy et al., blinded outcome evaluators do assess outcomes less optimistically than unblinded evaluators [39]. Thus, we tried to avoid unblinding of the external evaluators in our study. Consequently, they were not introduced in the therapeutic strategy at any time of the study to rule out that they would for example focus on a special subcategory of the psychological tests like phonologic memory. However as the evaluators in the case of the speech and intelligence tests directly interacted with the children and did see them five times within the course of the study, unblinding can not be ruled out.

Due to financial limitations, it was not possible for external therapists to conduct therapeutic music therapy ratings. Although the findings on inter-rater reliability in [18] give sufficient assurance on the reliability of the Nordoff-Robbins rating, an external rating would have probably been of higher internal validity with a more objective character.

Considering these limitations, our results should be interpreted with care.

### Underlying working principles

Until now, working principles of music therapy have mostly been examined using qualitative research. Although single case studies tried to identify possible modes of action in music therapy in speech development, we are still not able to isolate elements of music therapy as the driving therapeutic force. The only aspect that is for certain is that a mutually created musical dialogue improves the child's perception of him or herself and of the person who is sharing the experience. As a result, according to [27] the "activity of listening, in a structured musical improvisational context, without the lexical demands of language" may improve the cognitive, gestural, emotional, and relational development of the child.

Neuro-physiological approaches might underline these results but, due to limited resources, they have only been marginally applied in music therapy research. Additionally, it is questionable whether neuro-physiological approaches would be able to show development in areas such as social communication and self-consciousness, which are essential preconditions for language acquisition.

In addition, some important parameters for the development of language skills are not covered by the test parameters. Vital information was provided by the additional qualitative data collected from parents and testers. Most of the children started to use their language with increasing confidence; social skills improved and children were motivated to communicate more intensively. Although these data often did not correlate with the test results, these findings seem to be evidence of very important parameters that cannot be checked by the speech test and development tests alone. The Nordoff-Robbins scales, however, do reflect these aspects.

## Conclusions

Music therapy according to this study may have a beneficial effect on speech development. It does not seem to influence individual isolated aspects of speech development but might address and integrate many different aspects in a comprehensive way that are important for speech development, including relationship abilities and prosodic abilities. It might be supposed that music therapy interacts with very fundamental aspects of speech development and has measurable effects even after a short period of time. Therefore, music therapy may provide a very fundamental, basic, and supportive therapy for children with developmental speech delay.

## Competing interests

The authors declare that they have no competing interests.

## Authors' contributions

WF carried out the study, participated in the evaluation of data, and helped draft the manuscript. UL conceived of the study and carried out the study, participated in evaluation of data, and helped draft the manuscript. TO participated in the design of the study, performed the statistical analysis, and wrote the final version of the manuscript. All authors read and approved the final manuscript.

## Pre-publication history

The pre-publication history for this paper can be accessed here:

http://www.biomedcentral.com/1472-6882/10/39/prepub
